# Enhancing the Hydrolysis and Acyl Transfer Activity of Carboxylesterase DLFae4 by a Combinational Mutagenesis and In-Silico Method

**DOI:** 10.3390/foods12061169

**Published:** 2023-03-10

**Authors:** Longxiang Li, Liping Ding, Yuting Shao, Shengwei Sun, Mengxi Wang, Jiahui Xiang, Jingjie Zhou, Guojun Wu, Zhe Song, Zhihong Xin

**Affiliations:** 1 Key Laboratory of Food Processing and Quality Control, College of Food Science and Technology, Nanjing Agricultural University, Nanjing 210095, China; 2 Instrumental Analysis Center of CPU, China Pharmaceutical University, Ministry of Education, Nanjing 210009, China

**Keywords:** error-prone PCR, site-directed saturation mutation, molecular docking, acyltransferase activity, cyanidin-3-O-glucoside

## Abstract

In the present study, a feruloyl esterase DLFae4 identified in our previous research was modified by error-prone PCR and site-directed saturation mutation to enhance the catalytic efficiency and acyltransferase activity further. Five mutants with 6.9–118.9% enhanced catalytic activity toward methyl ferulate (MFA) were characterized under the optimum conditions. Double variant DLFae4-m5 exhibited the highest hydrolytic activity (270.97 U/mg), the *Km* value decreased by 83.91%, and the *Kcat/Km* value increased by 6.08-fold toward MFA. Molecular docking indicated that a complex hydrogen bond network in DLFae4-m5 was formed, with four of five bond lengths being shortened compared with DLFae4, which might account for the increase in catalytic activity. Acyl transfer activity assay revealed that the activity of DLFae4 was as high as 1550.796 U/mg and enhanced by 375.49% (5823.172 U/mg) toward 4-nitrophenyl acetate when residue Ala-341 was mutated to glycine (A341G), and the corresponding acyl transfer efficiency was increased by 7.7 times, representing the highest acyltransferase activity to date, and demonstrating that the WGG motif was pivotal for the acyltransferase activity in family VIII carboxylesterases. Further experiments indicated that DLFae4 and variant DLFae4 (A341G) could acylate cyanidin-3-O-glucoside effectively in aqueous solution. Taken together, our study suggested the effectiveness of error-prone PCR and site-directed saturation mutation to increase the specific activity of enzymes and may facilitate the practical application of this critical feruloyl esterase.

## 1. Introduction

Family VIII carboxylesterases represent a poorly characterized esterase family and are structurally more similar to class C β-lactamases than to carboxylesterases [1]. These proteins possess a Ser-Lys-Tyr catalytic triad, and their nucleophilic Ser is located in the S-X-X-K motif [2]. With the recent advance in metagenomics and molecular biotechnology, several new Family VIII carboxylesterases from different microorganisms have been isolated and identified [3,4], and most of them have been expressed and purified in fungi or bacteria [5]. They showed different coding sequences, protein structures, physicochemical properties, and catalytic activities. However, there were some drawbacks for most naturally derived Family VIII carboxylesterases, such as weak activity [6], poor thermal stability [7], and low yield [8], which greatly limited their application in practice. Directed evolution is a helpful way of improving enzyme properties for practical applications and a powerful tool for analyzing the relationship between enzyme structure and function [9,10,11,12]. A primary strategy for directed evolution is random mutagenesis, such as error-prone PCR [13], site-directed saturation mutation, DNA shuffling [14], and StEP recombination [15]. For example, the activity of endoglucanase Cel12A from *Thermotoga neapolitana* was improved by four times through four rounds of error-prone PCR [16]. A site-directed saturation mutation obtained a simvastatin synthase mutant G5 with a 4-fold increase in Kcat [17]. Directed evolution has become an effective and rapid method for improving enzyme properties by generating the desired mutants.

Recently, it was reported that several Family VIII carboxylesterases can catalyze the acetylation of nucleophiles with an acyl donor in an aqueous system, making them synthetically valuable while reducing the demand for expensive and difficult-to-handle organic solvents, representing a much more environmentally friendly and economical alternative to conventional chemical synthesis routes [18]. For example, *Pseudozyma antarctica* lipase A (CAL-A) can be used to synthesize fatty acid esters from natural oils and alcohols, even in the presence of bulk water [19]. MsAcT, an aryl esterase from *Mycobacterium smegmatis*, displayed unusual promiscuous acyltransferase activity and has been used to form various esters, carbonates, carbamates, and amides, including flavor compounds and high-value tryptamine derivatives [20]. EstCE1, belonging to family VIII of the esterase derived from a soil metagenome, catalyzes the irreversible amidation and carbamoylation of amines in water, which enables the synthesis of the antidepressant drug moclobemide from methyl 4-chlorobenzoate and 4-(2-aminoethyl) morpholine [21]. These findings stimulated a great effort to find more family VIII carboxylesterases with promiscuous acyltransferase activity [18]. However, only a few Family VIII carboxylesterases were found to catalyze acyl transfer in water efficiently and the number of carboxylesterases with strong activity is especially scarce. Therefore, there is still an increasing interest in searching for carboxylesterases with promiscuous acyltransferase activity for various applications.

In a previous study, a Family VIII carboxylesterase DLFae4 with a broad substrate range was identified and characterized from a soil metagenomic library [22]. DLFae4 can hydrolyze four methyl cinnamates, including methyl ferulate (MFA), methyl *p*-coumarate (M*p*CA), methyl caffeate (MCA), and methyl sinapate (MSA). In particular, the enzyme displayed robust hydrolytic activity as high as 123.78 U/mg towards MFA, which is extremely rare in previous reports [23,24].

This study used error-prone PCR and site-directed saturation mutation to construct a mutagenesis library. Five variants with enhanced hydrolytic activities toward MFA were obtained through two rounds of screening, followed by overexpression and biochemical characterization of the enzymes. The interactions between the receptor and ligand were analyzed by molecular docking. The acyltransferase activity of DLFae4 was characterized and improved. Strikingly, DLFae4 and variant DLFae4 (A341G) showed significant acyltransferase activity toward anthocyanin cyanidin-3-O-glucoside in aqueous solution.

## 2. Materials and Methods

### 2.1. Bacterial Strains, Plasmids, Enzymes, and Chemicals

Plasmid pET28a (+) (Takara, Dalian, China) and *Escherichia coli* BL21 (DE3) Competent Cells (Vazyme, Nanjing, China) were used as an expression vector and host for recombinant protein, respectively. Restriction enzymes (*NcoⅠ* and *XhoⅠ*), T4 DNA ligase, Taq DNA polymerase, deoxy-ribonucleoside triphosphate (dATP, dGTP, dCTP, and dTTP), and MgCl_2_ were obtained from Takara (Dalian, China), and MnCl_2_ was purchased from Sinopharm (Beijing, China). Oligonucleotide primers synthesis and sequencing services were provided by General (Chuzhou, China) corporation. Other reagents were purchased from Macklin (Shanghai, China).

### 2.2. Construction of Random Mutagenesis Library

Error-prone PCR was carried out for the first round of mutation. The recombinant plasmid pET-*DLFae4* was used as the template for error-prone PCR, and the primers include FAE-F 5′-CATGCCATGGACGTGACCACCACGAT-3′ and FAE-R 5′-CCGCTCGAGTACACTCGCATACACC-3′ (underlined bases were *NcoI* and *XhoI* sites, respectively). A 50 μL PCR system contained 0.2 mM dATP and dGTP, 0.8 mM dCTP and dTTP, 3 mM Mg^2+^, 0.1~0.5 mM Mn^2+^, 0.4 μM of each primer, 5 μL 10 × Taq buffer, 5U Taq DNA polymerase, and 20 ng template plasmid. The PCR conditions include initial denaturation at 94 °C for 5 min, followed by denaturing at 94 °C and 30 s for 30 rounds, annealing at 55 °C for 30 s, extension at 72 °C for 1.2 min, and a final extension at 72 °C for 10 min. The PCR product was cloned into the pET28a(+) vector by restriction endonuclease *NcoI*, *XhoI*, and T4 DNA ligase. Then the recombinant plasmids with mutated genes of DLFae4 were transformed into *E. coli* BL21 (DE3) cells and the transformants were grown on agar-stabilized lysogeny broth (LB) plates containing 50μg/mL Kanamycin (Kana) overnight. The mutant library was prepared in 20% (*v*/*v*) sterilized glycerol and stored at −80 °C.

### 2.3. Construction of Site-Directed Saturation Mutagenesis Library

The most active variant DLFae4-m2 obtained by Error-prone PCR was used as a template, and the mutation sites were designed by the HotSpot Wizard server (Table S1). The site saturation mutagenesis library was constructed by a Site-directed Mutagenesis Kit (Sangon Biotech, Shanghai, China). The recombinant plasmids pET28a(+)-*DLFae4-m2* were used as the template, and the degenerate primers (145F 5′-TGACAAACVDSCCTGGTATCGTTTCCG-3′, 145R 5′-CGGAAACGATACCAGGSHBGTTTGTCA-3′, 341-F 5′-TAGGAAGTTGTTGCTGGGGTGGCCTATTCAACAGC-3′, 341-R 5′-TGAATAGGCCACCCCAGCAACAACTTCCTACTGC-3′; underlined bases were degenerate codons) were used to amplify the whole plasmid.

### 2.4. Screening of Mutant Libraries

Mutants and original clones were transferred to screening plates that contained 0.15% (*m*/*v*) ethyl ferulate and 50 μg/mL Kana and incubated at 37 °C for ten hours. The activity of clones was identified preliminarily by the larger size of the halos around the clones. The positive mutants were rescreened by detecting the enzymatic hydrolysate ferulic acid (FA) using high-performance liquid chromatography (HPLC) (LC-20A system, Shimadzu, Kyoto, Japan). The purified enzyme was added to 100 Mm Tris-HCl buffer (pH 8.6) with 1 mM MFA at 60 °C for 3 min, and the reaction was terminated by boiling for 5 min. HPLC was carried out on a Zorbax SB-C18 column at 30 °C, employing water containing 1% acetic acid as solvent A and methanol as solvent B at a rate of 0.5 mL/min. The identified positive mutants were confirmed by sequencing.

### 2.5. Expression and Purification of DLFae4 and Mutants

Positive clones were incubated in a 5 mL LB broth medium containing 50 μg/mL Kana at 37 °C overnight. Subsequently, the solution was transferred to a 200 mL LB broth medium containing 50 μg/mL Kana with 1% (*v*/*v*) volume and incubated at 37 °C. When the OD_600_ reached 0.6–0.8, isopropyl-β-D-thiogalactopyranoside (IPTG) was added to a final concentration of 0.5 mM to induce the expression of recombinant enzymes. After being cultured at 16 °C for 20 h, the cells were harvested by centrifugation (4 °C, 10,000× *g*, 20 min) and resuspended in potassium phosphate buffer (50 mM NaH_2_PO_4_ and 300 mM NaCl, pH 8.0). Then the cells were disrupted by sonication (SCIENTZ-IID, SCIENTZ, Ningbo, China), and the supernatant was collected as a crude enzyme by centrifugation (4 °C, 12,000× *g*, 20 min). The crude enzymes were purified by Ni-NTA-Sefinose column (Sangon Biotech, Shanghai, China) according to the manufacturer’s instructions. The concentrations of purified enzymes were determined by a Micro-Volume UV–Vis spectrophotometer (NanoDrop, Thermo Scientific Co., Ltd., Waltham, MA, USA), and the molecular masses were determined by sodium dodecyl sulfate-polyacrylamide gel electrophoresis (SDS-PAGE).

### 2.6. Characterization of DLFae4 and Mutants

The effect of pH on enzyme activity was measured at 37 °C in various buffers: pH 5–7, 200 mM Na2HPO4 and 100 mM citric acid buffer, pH 8–8.6, 100 mM Tris-HCl buffer, and pH 9.6–10.6, 200 mM glycine-NaOH buffer. The maximal activity was defined as 100%, and the relative activity is shown as a percentage of maximal activity. To determine pH stability, the enzyme was incubated at various pH levels for 1 h at 4 °C, and residual activity was measured after adding substrate. The optimum temperature was determined in a range of 20 to 70 °C at the optimum pH. For the thermostability determination, the enzyme was incubated at 60 °C for 10–50 min, and residual enzyme activity was measured under optimum conditions after each incubation time. The nonincubated enzyme was taken as the control, and all reactions were conducted in triplicate for statistical analysis.

### 2.7. Calculation of Enzyme Kinetic Parameters

Enzyme reactions were performed using 0.025–2 mM MFA in 100 mM Tris-HCl buffer (pH 8.6) at 60 °C, incubating for 3 min, and was terminated by boiling for 5 min. The esterase activity of DLFae4 was determined by detecting the released FA from the hydrolysis of MFA using HPLC with a detection wavelength of 320 nm. One unit of esterase activity was defined as the amount of enzyme required to release 1 μmol of FA from MFA per min. Enzyme kinetic parameters (*Km* and *Kcat*) were calculated according to the Lineweaver–Burk plot. All reactions were conducted in triplicate for statistical analysis, and all data are the averages from three independent experiments.

### 2.8. Structural Modeling and Molecular Docking

AlphaFold 2.0 (https://alphafold2.biodesign.ac.cn/, accessed on 10 October 2022) was used to model three-dimensional protein structures. AutoDockTools1.5.6 performed the molecular docking, and the docking result was visualized by PyMOL and analyzed by Discovery Studio.

### 2.9. Multiple Sequence Alignments of DLFae4

Multiple sequence alignments were performed by CLUSTALW (https://www.genome.jp/tools-bin/clustalw, accessed on 12 October 2022) and ESPript 3.0 (https://espript.ibcp.fr/ESPript/cgi-bin/ESPript.cgi, accessed on 12 October 2022).

### 2.10. Identification and Characterization of the Acyltransferase Activity

For the initial identification, a monophasic and clear reaction mixture (200 mM vinyl acetate and 20 mM benzyl alcohol in 200 mM potassium phosphate buffer, pH 8.0) was transferred to an EP tube (950 μL per well), and 50 μL of the purified enzyme was added to the reaction mixture. The reaction mixtures were incubated at room temperature (25 °C), and the formation of emulsions was observed by the naked eye, indicating the promiscuous acyltransferase activity.

To further identify the reaction product, the mixtures were extracted with 500 μL methyl tert-butyl ether (MTBE), rapidly vortexed, and then centrifuged to separate the phases (12,000× *g*, 1 min). The organic phases were dried over anhydrous sodium sulfate prior to analysis by gas chromatography (GC). A 7890GC/5975MSD GC–MS (Agilent Technologies Inc., Santa Clara, CA, USA) coupled with an HP-5 column was used, and the GC–MS conditions were as described previously [18]. The product’s chemical structure was identified by comparison with the data in the database NIST08.

In order to test the acyltransferase activity of DLFae4 and variant DLFae4 (A341G), reactions for benzyl acetate formation contained purified DLFae4 or variant DLFae4 (A341G) (0.1 mg/mL), 50 mM benzyl alcohol, and 200 mM vinyl acetate in 1 mL of 200 mM potassium phosphate buffer (pH 8.0). Time samples (50 µL) were taken after 5, 10, 15, 20, 25, 30, 40, 60, 90, 130, and 180 min. Reactions for benzyl methyl carbonate formation contained purified enzyme, 20 mM benzyl alcohol, and 200 mM dimethyl carbonate in 1 mL of 200 mM potassium phosphate buffer (pH 8.0). Time samples (50 µL) were taken after 5, 10, 15, 20, 25, 30, 40, 60, 90, 130, 180, 240, and 480 min. The pretreatment of samples and GC-MS analysis were the same as the description above.

As in a previous report, the acyltransferase activity of the enzyme was determined by *p*-nitrophenyl acyltransferase (*p*NP-AcT) assay [18]. Reactions containing benzyl alcohol (0, 0.05, 0.1, 0.5, 1, 2.5, 5, 10, 15, 20, 25 or 30 mM), 1.8 mM *p*NP-ester (*p*NP-C2, *p*NP-C4, *p*NP-C6, and *p*NP-C8) and purified enzymes in 200 mM potassium phosphate (pH 8.0) were conducted in 200 µL scale in 96-well microtiter plates at 25 °C, Varioskan Flash multi-function microplate reader (Thermo Fisher Scientific, Waltham, MA, USA) was used to measure the absorbance value at 405 nm. Specific acyl transfer activity (AT) was determined by analyzing the released quantity of *p*-nitrophenolate and compared with reaction systems without benzyl alcohol. All reactions were conducted in triplicate, and negative controls were performed by subtracting *p*NP-esters background interference in the buffer.

Specific hydrolysis activity (H) was determined by reactions containing 2, 1, 0.5, 0.3, 0.2, 0.1, 0.05, or 0.025 mM *p*NP-ester and 0.5 mg/mL purified enzymes in 200 mM potassium phosphate (pH 8.0). The reaction conditions and measurement methods were the same as described in the *p*NP-AcT assay.

### 2.11. Acylation of Cyanidin-3-O-Glucoside

The reaction was performed in 200 mM potassium phosphate buffer (pH 8.0) containing 2.5 mM vinyl acetate, 0.5 mM cyanidin-3-O-glucoside, and 0.5 mg/mL purified enzymes. The reactions were incubated at 40 °C for 10 min. An ultrahigh-performance liquid chromatography-electrospray ionization-tandem mass spectrometry (UPLC-ESI-MS/MS) system (G2-XS QTof, Waters Milford MA USA) was used to analyze the molecular mass of the product. Three microliters of the solution was injected into the UPLC column (2.1 × 100 mm ACQUITY UPLC BEH C18 column containing 1.7 μm particles) with a flow rate of 0.3 mL/min. Mobile phase A was water containing 0.5% formic acid, while mobile phase B was acetonitrile. The gradient program of the mobile phase was as follows: 0–1 min, 5% B; 1–5 min, 5–90% B; 5–6 min, 90% B; 6–6.1 min, 90–5% B; 6.1–8 min, 5% B. UPLC-ESI-MS/MS was performed with a collision energy of 20 eV employing an electrospray source in positive ion mode with a selected mass range of 50–600 m/z. The ionization parameters included the following: capillary voltage of 3.0 kV, cone voltage of 40 V, source temperature of 550 °C, and dissolution gas temperature of 400 °C. Data acquisition and processing were performed employing Masslynx 4.1 (Waters, Milford, MA, USA) software package.

### 2.12. Statistical Analysis

The data are presented as the mean ± standard deviation (SD, *n* = 3). Statistical analyses were performed using the Microsoft Excel software package.

## 3. Results

### 3.1. Construction of Random Mutagenesis Library and Site-Saturation Mutagenesis Library and Screening

A mutant library with about 10,000 clones was constructed by error-prone PCR. Preliminary screening was performed according to the size of the halos around the colony using ethyl ferulate (EFA) as substrate (Figure S1a). Three mutants (DLFae4-m1~m3) with 21.3%, 35.7%, and 6.9% increases in hydrolytic activity were identified (Table 1, Figures S1 and S2) after the second screening by HPLC analysis.

Variant DLFae4-m2 exhibited the strongest hydrolytic activity and was used as a template targeting position T145 for site-saturation mutagenesis. The mutation site was predicted by HotSpot Wizard, which is an interactive web server used for the automated identification of hotspots, resulting in mutants with improved protein stability, catalytic activity, substrate specificity, and enantioselectivity [25] After the screening, two variants DLFae4-m4 and DLFae4-m5, with further improved hydrolytic activity, were acquired. Their activity was increased by 54.7% and 118.9% (Figures S1 and S2, Table 1) than that of WT, respectively.

### 3.2. Expression and Purification of Recombinant Enzymes

**Figure 1:**
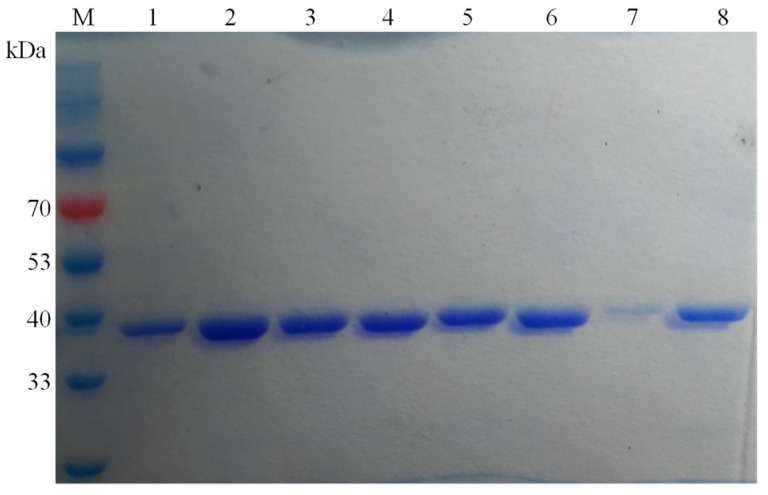
SDS-PAGE analysis of DLFae4 and variants. lane 1, purified DLFae4 protein; lane 2, purified variant DLFae4-m1 protein; lane 3, purified variant DLFae4-m2 protein; lane 4, purified variant DLFae4-m3 protein; lane 5, purified variant DLFae4-m4 protein; lane 6, purified variant DLFae4-m5 protein; lane 7, null; lane 8, purified variant DLFae4 (A341G) protein; lane M, standard protein marker.

DLFae4 and variant genes with a histidine tag at the C-terminus were integrated into a pET28a (+) vector containing a strong T7 promoter, and transformed into *E. coli* BL21 (DE3) for overexpression. The recombinant enzyme was purified through affinity chromatography with a Ni-NTA column. SDS-PAGE was used to examine enzymes’ purity and molecular mass, and the result showed that both DLFae4 and variants gave a single clear band with a molecular mass of approximately 38.3 kDa (Figure 1).

### 3.3. Biochemical Characterization of DLFae4 and Variants

To gain a deeper understanding of the variants DLFae4-m1~m5, their activity and stability were determined at different temperatures and pH levels, as shown in Figure S3. The optimal temperature of DLFae4 and variants was 60 °C, the residual activities of the five variants remained above 50%, and DLFae4 remained above 46% (Figure S3a), indicating that the variants are more heat-resistant than WT. The thermostability experiment revealed that DLFae4-m4 and DLFae4-m5 maintained more than 60% residual activity after 50 min incubation at 40 °C, while the activity of DLFae4 declined below 40% (Figure S3c), suggesting that the variants showed better thermal stability than DLFae4.

The effect of pH on the activity of DLFae4 and variants were investigated over the pH range of 5–11, and the enzymes exhibited maximal activity at pH 8.6. Fifty percent relative activity remained at pH 10.6, while the relative activity was less than 20% at pH 5 (Figure S3b). The effect of pH on the stability of enzyme activity was determined after 60 min incubation, and the relative activity of variants was comparable to DLFae4 (Figure S3d). In particular, DLFae4-m5 retained the highest activity among variants when pH was 8.6–10.6, even maintained 70% residual activity after the incubation at pH 10.6, while the others all retained about 55%, indicating that DLFae4-m5 had a stronger alkali-tolerance than DLFae4 and other variants.

### 3.4. Enzymatic Kinetic Parameters of DLFae4 and Variants

To further investigate the catalytic efficiency of the five variants, kinetic parameters were calculated following Michaelis–Menten equation using MFA as substrates (Table 1). The reaction rates were initially tested before fitting into the Lineweaver–Burk plots by Excel software package. All variants exhibited a higher substrate affinity and a more effective catalytic efficiency toward MFA than that WT. Specifically, variant DLFae4-m5 showed the most robust catalytic activity among all variants with a 118.9% increase, while the *Km* value decreased by 83.91% and the *Kcat/Km* value improved by 6.08-fold.

### 3.5. Structural Modeling and Molecular Docking

In order to gain a deeper insight into the mechanism of the activity-enhanced variants, structural modeling and molecular docking of the enzymes was performed. The three-dimensional structures of the enzymes were predicted with AlphaFold, which is currently the top-ranked protein structure prediction approach with unprecedented accuracy [26,27]. The models were evaluated by analyzing Ramachandran plots. In theory, a good quality model would be expected to have over 90% residues in the most favored regions. The results showed that the percentages of WT and variants in the most favored regions were more than 95% (Figure S4), indicating that the constructed model is highly reliable. Molecular docking was performed using the 3D structure of DLFae4 as the receptor and MFA as the ligand by Autodock 4.2. Pymol (version 2.4.1) was applied to visualize 3D models. The conformations with the lowest free energy from the docking results were selected to analyze the protein-ligand interactions and speculate the possible mechanisms of activity enhancement.

**Figure 2:**
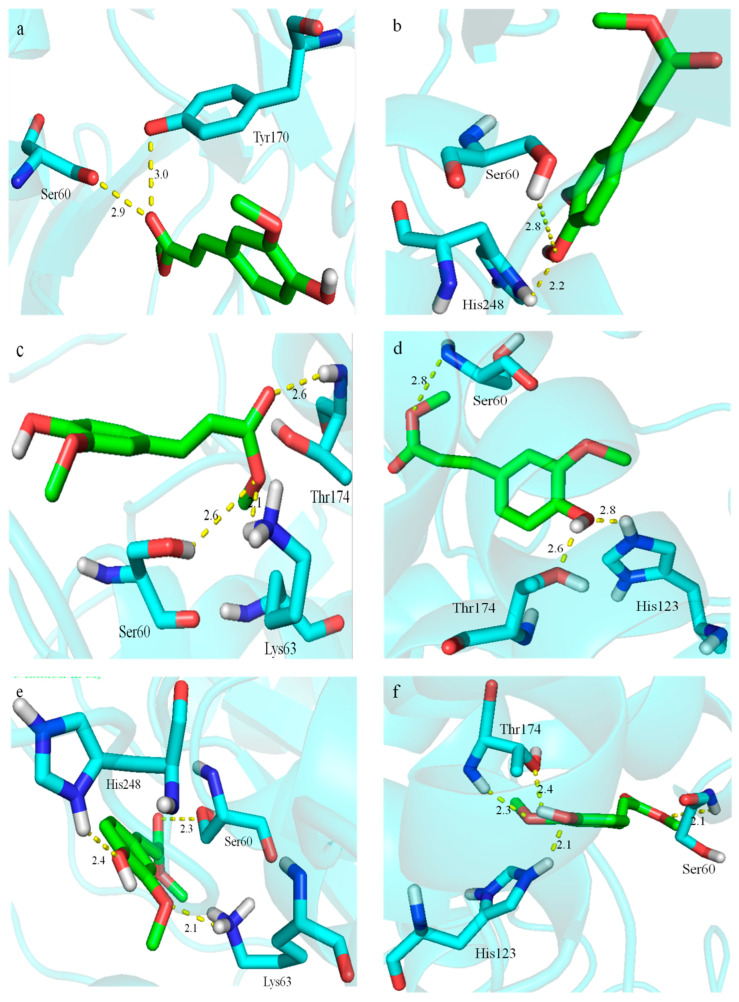
The hydrogen bond interaction between the active site of the enzyme and MFA. (**a**): DLFae4; (**b**): DLFae4-m1; (**c**): DLFae4-m2; (**d**): DLFae4-m3; (**e**): DLFae4-m4; (**f**): DLFae4-m5. The substrate MFA is represented by a green stick pattern, the amino acid residues that form hydrogen bonds with MFA are represented by a cyan stick pattern, and the hydrogen bonds are represented by a yellow dashed line.

Hydrogen bonding is one of the main interactions to determine the binding strength of an enzyme and substrate. Generally, the stronger the reactions between an enzyme and substrate, the more numbers and the shorter the length of hydrogen bonds. The docking results revealed that MFA-CO established two hydrogen bonds with Ser60-OH and Tyr170-OH with lengths of 2.9 Å and 3.0 Å in DLFae4 (Figure 2a). For DLFae4-m1, Ser60-OH and His248-NH formed two hydrogen bonds with MFA-Ph-OH with lengths of 2.8 Å and 2.2 Å, reduced by 0.1 Å and 0.7 Å, respectively. Accordingly, the enzymatic activity increased by 21.29% as compared with DLFae4 (Figure 2b), indicating that the bond length of hydrogen bonds had an essential effect on the improvement of enzymatic activity.

In variants DLFae4-m2~DLFae4-m4, a hydrogen bonds network was formed among three amino acid residues of the enzymes and substrate MFA. For DLFae4-m2, Ser60-OH and Lys63-NH_3_ generated two hydrogen bonds (2.6 Å and 2.1 Å) with MFA-CH_3_O, Thr174-NH, and MFA-CO generated the third hydrogen bond with a length of 2.6 Å (Figure 2c), and the catalytic activity improved by 35.75%. In DLFae4-m3, two hydrogen bonds (2.6 Å and 2.8 Å) were formed by Thr174-OH and His123-NH with MFA-Ph-OH, Ser60-NH created a 2.8 Å hydrogen bond with MFA-CH_3_O (Figure 2d), the catalytic activity increased by 6.9%. This might be because the hydrogen bond between Ser60-NH and MFA-CH_3_O only shortened by 0.1 Å, resulting in only a slight increase in the catalytic activity of the enzyme. In DLFae4-m4, His248-NH and MFA-Ph-OH and Lys63-NH_3_ and MFA-Ph-CH_3_O created two hydrogen bonds (2.4 Å and 2.1 Å), the third hydrogen bond formed by Ser60-OH and MFA-CO with a length of 2.3 Å (Figure 2e), while the catalytic activity improved by 54.66%, which is probably because these three hydrogen bonds were much more potent than WT. In particular, the bond length between Ser60-OH and MFA-CO was reduced by 0.6 Å. Thus, it increased the enzyme’s catalytic activity significantly, suggesting that the type, number, and bond length of hydrogen bonds have a critical effect on the interactions between enzyme and substrate, which might lead to the increased catalytic activity of variants.

For DLFae4-m5, a complex hydrogen bond network was formed by three amino acid residues and MFA. MFA-Ph-CH_3_O and Thr174-NH created a hydrogen bond with a length of 2.3 Å. MFA-Ph-OH generated two hydrogen bonds with Thr174-OH and His123-NH with lengths of 2.4 Å and 2.1 Å, respectively. Ser60-NH formed the fourth hydrogen bond (2.1 Å) with MFA-CH_3_O (Figure 2f). The complex hydrogen bond network enhanced the interactions of enzyme and substrate and improved the catalytic activity of DLFae4-m5 by 118.9%.

An analysis of surface hydrophobicity showed that the hydrophobic pocket and surface hydrophobic amino acid residues of variants differed from that of WT (Figure S5). Most of the MFA in DLFae4-m1~DLFae4-m3 was exposed to the outside of the hydrophobic pocket, while MFA in variants DLFae4-m4 and DLFae4-m5 was almost completely embedded in the hydrophobic pocket, indicating the latter had a stronger hydrophobic interaction than the former and was more likely to react with MFA.

### 3.6. Preliminary Identification of Acyltransferase Activity

An acyltransferase activity experiment was carried out using 2-phenylethanol and vinyl acetate as substrates to identify acyltransferase activity. The occurrence of turbidity in aqueous solution resulted from 2-phenylethyl acetate, which is the reaction product with low water solubility and was further confirmed by GC-MS (Figures S6 and S7), indicating that DLFae4 exhibited promiscuous acyltransferase activity with a preference for aromatic acyl-acceptor substrates and was consistent with the result in the literature [18].

### 3.7. Site-Directed Mutagenesis and Characterization of the Acyltransferase Activity

**Figure 3:**
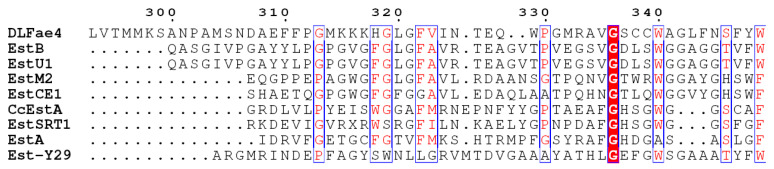
Multiple sequence alignment of DLFae4 and other closely related VIII family esterases.

**Figure 4:**
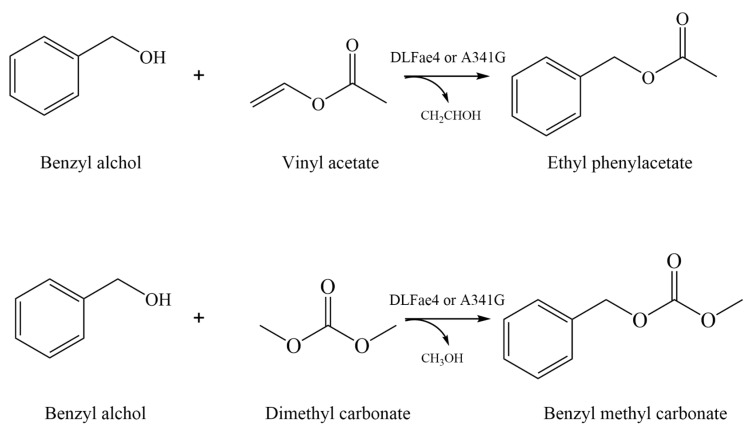
Enzymatic synthesis of esters and carbonates by DLFae4 or variant DLFae4 (A341G).

It was reported that a WGG motif played an important role in the acyltransferase activity in family VIII carboxylesterases [18]. By mutation of the HDG of family VIII carboxylesterases 3ZYT to HGG or WGG, Henrik Müller et al. rationally transformed a hydrolase into an acyltransferase and demonstrated the importance of WGG motif in the acyl transfer reaction [18]. In the current study, multiple sequence alignment of DLFae4 with closely related family VIII carboxylesterases was performed (Figure 3). The result revealed that the DLFae4 protein sequence had a WAG motif at positions 340–342 corresponding to the position of the WGG motif of enzymes in this family. Therefore, the alanine at the 341 site was mutated to glycine (A341G), and the acyltransferase activity of DLFae4 and DLFae4 (A341G) was tested in aqueous solution (Figure 4).

**Figure 5:**
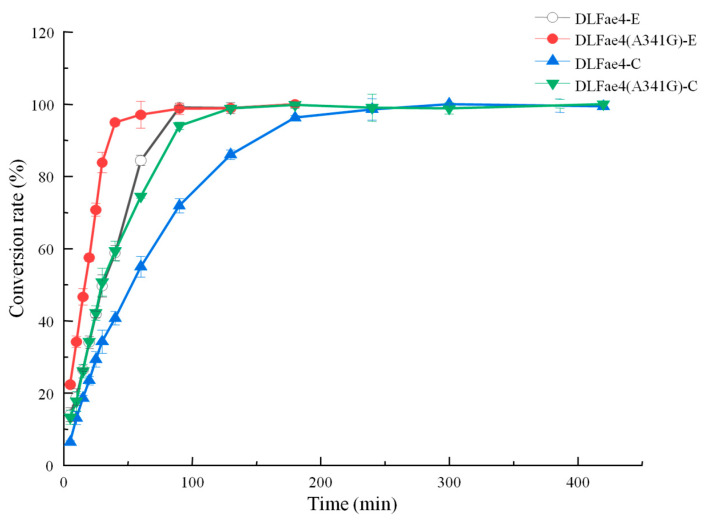
DLFae4 and A341G catalyze the synthesis of benzyl acetate (circle) and benzyl methyl carbonate (triangle) in phosphate buffer. DLFae4-E: the conversion of benzyl acetate by DLFae4, DLFae4 (A341G)-E: the conversion of benzyl acetate by variant DLFae4 (A341G), DLFae4-C: the conversion of benzyl methyl carbonate by DLFae4, DLFae4 (A341G)-C: the conversion of benzyl methyl carbonate by variant DLFae4 (A341G). Note: All assays were performed in triplicate, and the values shown are mean ± standard deviation.

Results showed that esters and carbonates were synthesized by DLFae4 and DLFae4 (A341G) with different reaction rates (Figure 5). The conversion rates of esters and carbonates were gradually increased with the extension of reaction time, and no product hydrolysis was detectable even after 12 h, indicating all the reactions appear to be irreversible. The benzyl acetate formation from benzyl alcohol and vinyl acetate was very fast, with more than 90% conversion rate within 50 min, and the reaction time decreased by 30 min as compared with variant DLFae4 (A341G) and DLFae4. The benzyl methyl carbonate formation from benzyl alcohol and dimethyl carbonate was slower than that of benzyl acetate. The highest conversion rate of variant DLFae4 (A341G) was accomplished within 120 min while DLFae4 required an extra 80 min as achieved the same conversion rate, indicating the faster reaction rate and stronger transacylase activity of variant DLFae4 (A341G).

In order to determine the specific acyltransferase activity of DLFae4 and variant DLFae4 (A341G), the *p*NP-AcT assay was carried out using *p*NP-ester of different chain-lengths (C2 to C8) as acyl donor and benzyl alcohol as acceptor. The acyl transfer efficiency of DLFae4 and variant DLFae4 (A341G) was characterized by the AT:H ratio (acyltransferase activity: hydrolase activity) (Table 2). The result suggested that DLFae4 and variant DLFae4 (A341G) proved to be valuable for the acylation of benzyl alcohol with chain lengths ranging from C2 to C8. Notably, variant DLFae4 (A341G) exhibited a remarkable acyltransferase activity as high as 5823.172U/mg towards 4-nitrophenyl acetate (*p*NP-C2), representing the most vigorous acyltransferase activity to date. Furthermore, the specific acyl transfer activity of variant DLFae4 (A341G) toward pNP-C2 and pNP-C4 were enhanced by 375.49% and 75.8%, while the acyl transfer efficiency (AT:H) was increased by 7.7 and 3.2 times than that of WT, respectively.

### 3.8. Acylation of Cyanidin-3-O-Glucoside

**Figure 6:**
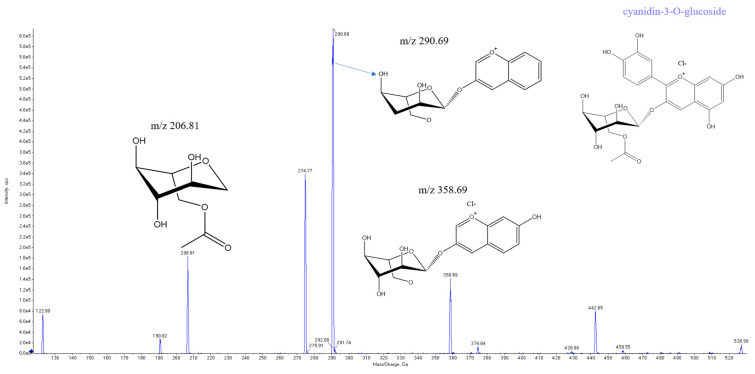
Tandem MS/MS spectrometry for the acetylation product of cyanidin-3-O-glucoside.

Anthocyanins, a group of water-soluble natural pigments, are widely used in food and biotechnology for their antioxidant, cytoprotective, and neuroprotective properties [28]. To explore the wider application of the acyltransferase activity of DLFae4 and variant DLFae4 (A341G), an acylation reaction was performed by cyanidin-3-O-glucoside as acyl acceptor and vinyl acetate as an acyl donor. When catalyzed by DLFae4 and variant (A341G), a new peak appeared at 22 min at a wavelength of 520 nm by HPLC-DAD analysis, which was presumed to be the acylated product of cyanidin-3-O-glucoside (Figure S8). Detection of the ion fragments indicated that a [M+H]+ ion at m/z 526.99 was speculated to be a molecular formula of C_13_H_23_ClO_12_. A conjugate of cyanidin-3-O-glucoside with an acetyl moiety attached was identified according to the signals of m/z 206.81, 290.69, and 358.69 in the MS/MS spectrum of the parent ion m/z 526.99 (Figure 6). The results were consistent with the findings by previous studies that enzymatic acylation of glucoside generally happened at the 6″-OH site of glucose [29], suggesting that cyanidin-3-O-glucoside was acylated successfully by DLFae4 and variant DLFae4 (A341G) at the same position.

## 4. Discussion

Our previous investigation identified a new feruloyl esterase DLFae4 from a soil metagenomic library with strong hydrolytic activity toward MFA. For a deeper understanding of the repertoire and applying DLFae4 in practice, directed evolution was utilized to improve the catalytic performance further and explore the possible acyl transfer activity.

Directed evolution of enzymes has emerged as a powerful tool in identifying enzyme variants with new or improved properties through a simulated natural selection process in the lab, like enhancing activity [30], thermostability [31] and acid stability [32], and the evolutionary process could be accomplished in a few weeks or months instead of hundreds of years in nature. Error-prone PCR, the most popular method to introduce random mutations during PCR by reducing the fidelity of the DNA polymerase, has been widely applied in synthetic biology and protein modification [10]. For example, by constructing and screening, Gabriella Cerullo et al. improved by 74.6% the catalytic activity against MFA of a type C feruloyl esterase FoFaeC from *Fusarium oxysporium* using a library of around 30,000 random mutants generated by error-prone PCR [33].

Another important strategy for directed evolution is site-directed saturation mutation, which designs degenerate primers at defined sites in a pre-determined way, thus allowing the substitution of one or more specific sites against all possible mutations in a gene sequence. In some cases, it is used to clarify the relationship between protein structure and properties and is considered a powerful tool to modify DNA sequences in molecular biological studies and genetic engineering [34]. Haowen Zhang et al. rationally increased 4- and 5-fold catalytic efficiency of a feruloyl esterase LP_0796 from *Lactobacillus plantarum* by mutation Asp with Ala23 and Ile198, respectively [35]. Eight mutants were generated based on the simulated structure to enhance the catalytic efficiency of a feruloyl esterase FAELac, among which Q198A and Q134T were enhanced by 5.4- and 4.3-fold catalytic efficiencies, respectively, in comparison with WT [36]. In this study, to improve the activity of DLFae4 to an extreme, error-prone PCR and site-directed saturation mutation was applied combined with an in-silico method, and five variants with enhanced catalytic activity, including DLFae4-m1 (K216R), DLFae4-m2 (F156L), DLFae4-m3 (S299T), DLFae4-m4 (F156L&T145L) and DLFae4-m5 (F156L&T145M) were identified, and the best variant DLFae4-m5 increased hydrolytic activity by 118.9% compared to WT.

Molecular docking, an attractive bioinformatic tool utilized to predict the predominant binding mode(s) of a ligand with a protein of known three-dimensional structure, places ligands into the preferred binding sites of specific regions of protein receptor targets. The complex obtained from the docking facilitated an understanding of the potential binding affinity and the possible mechanism of action between the ligand molecule and the target. The present study showed that one hydrogen bond was formed between the Ser60 of enzymes and the ester carbonyl group or the ester oxygen atom of MFA. For variants DLFae4-m1~DLFae4-m5, the hydrogen bond lengths between Ser60 and MFA were shorter than that of WT, indicating stronger hydrogen bonds were formed in these variants. Ser60 is an indispensable amino acid residue that completes the catalytic triad and played an essential role in the nucleophile attack toward a substrate. Studies have shown that changes in the distance between Ser60 and the ester bond of the substrate would affect the enzymatic catalytic efficiency [37]. When the distance from Ser to the substrate carbonyl ester shortened, nucleophilic reactions were more likely to occur, and the formation of mesotetrahedrons was induced, thereby increasing catalytic efficiency and enhancing the enzymatic activity of variants.

Promiscuous acyltransferases are able to efficiently catalyze acyl transfer in monophasic aqueous media, thereby overcoming the requirement for expensive organic solvents, harsh reaction conditions, and long reaction times. Promiscuous acyltransferase activity has been found in several types of hydrolases, such as esterase [38], lipases [19,39], and carboxylesterases [40]. Since the discovery of MsAcT, the first promiscuous acyltransferase with remarkable transfer efficiency in the presence of a large amount of water from *Mycobacterium smegmatis* in 2007 [18,41], much effort has been directed toward excavating further promiscuous acyltransferases. Lukas Reisky established a highly effective ultra-high-throughput acyltransferase assay and used it for mining a metagenome-derived promiscuous acyltransferase Est8, the first reported member from the bacterial hormone-sensitive lipase(bHSL) family [38]. Based on the structure of Est8, a scheme for sequence-based prediction of promiscuous acyltransferase activity was established and led to the discovery and characterization of five more acyltransferases. These findings imply that there must be a large number of unexcavated acyltransferases in nature [40].

However, only a few promiscuous acyltransferases with similar acyl transfer activity in the presence of bulk water have been exploited and characterized in previous studies. In particular, the number of acyltransferases with strong activity was very limited, which greatly hampered the broad application of enzymes in industry. In the present study, the activity of promiscuous acyltransferase DLFae4 was as high as 1550.796 U/mg toward pNP-C2, and further improved by 375.49% (5823.172 U/mg) when the WAG motif at the 341 site was mutated to the WGG motif, the corresponding AT:H were increased by 7.7 times compared to that of WT, representing the highest acyltransferase activity to date. The activity of promiscuous acyltransferase of the variant was almost equal to the acyltransferase activity of EstM2 (5583 U/mg) [18] with a WGG motif, suggesting that the particular motif plays a critical role in the acyl transfer activity in the family VIII esterases, which was consistent with the previous report.

It has been demonstrated that the WGG motif was pivotal for the acyltransferase activity in family VIII carboxylesterases, in which tryptophan is largely helpful for the formation of the hydrophobic cavity and promotes the binding and positioning of organic nucleophiles [18]. For example, an esterase EstA with HDG sequence showed no acyltransferase activity but exhibited significant activity when the HDG motif mutated to HGG or WGG. On the contrary, the WGG motif in EstCE1 from family VIII carboxylesterase, which exhibited remarkably acyltransferase activity in kU/mg enzyme, was mutated to WDG, resulting in the AT:H ratio decreasing sharply. The results of the present study further confirmed that the WGG motif is critical to the acyltransferase activity in family VIII carboxylesterases. In addition, cyanidin-3-O-glucoside was acylated successfully by DLFae4 and DLFae4 (A341G) using vinyl acetate as the acyl donor and cyanidin-3-O-glucoside as the acyl acceptor, illustrating the important role of promiscuous acyltransferases in protecting natural pigments, which are unstable and easy to fade when exposing to harsh circumstances during food processing and storage [28], providing a more efficient and economical option for the acylation of anthocyanins.

## 5. Conclusions

In summary, error-prone PCR and site-directed saturation mutation were used to improve the hydrolytic activity of family VIII carboxylesterase DLFae4. Five variants with enhanced hydrolytic activities were identified, and molecular docking elucidated the possible reason for the enzymes’ enhanced activity. The promiscuous acyltransferase activity of DLFae4 was determined by pNP-AcT assay enhanced extraordinarily by mutating the WAG motif to the WGG motif. The WT and variant successfully catalyze the acylation reaction of cyanidin-3-O-glucoside. The results from the current investigation indicated that error-prone PCR and site-directed saturation mutation combined in-silico is a useful pipeline to improve the activity of enzymes, providing new insight for the application of family VIII carboxylesterase in industry. Further investigation will be carried out to uncover the mechanism of the hydrolysis and acyl transfer activity and insights into drug metabolism and pharmacokinetics information in depth.

## Figures and Tables

**Table 1:** Kinetic parameters of DLFae4 and variant on MFA.

Enzyme	Specific Activity (U/mg)	*Km* (mM)	*Kcat* (S^−1^)	*Kcat*/*Km* (Mm^−1^ s^−1^)
DLFae4	123.78 ± 3.23	0.23 ± 0.012	79.01 ± 2.06	343
DLFae4-m1	150.13 ± 2.05	0.086 ± 0.011	37.27 ± 1.02	429
DLFae4-m2	168.03 ± 1.19	0.051 ± 0.013	60.24 ± 0.46	1172
DLFae4-m3	132.33 ± 2.24	0.069 ± 0.008	46.95 ± 1.23	675
DLFae4-m4	191.44 ± 0.78	0.047 ± 0.010	77.36 ± 0.06	1612
DLFae4-m5	270.97 ± 0.95	0.037 ± 0.002	79.10 ± 0.07	2089

Note: Using MFA as substrate. Data were shown as mean ± standard deviations with triplicate replicates.

**Table 2:** Transfer efficiency of DLFae4 and A341G to acyl donors of different chain lengths.

Enzyme	Acyl Doner	AT in U/mg	Hydrolysis in U/mg	AT:H
DLFae4	pNP-C2	1550.796 ± 53.98	91.135 ± 7.57	17.016
	pNP-C4	232.218 ± 18.93	71.009 ± 3.21	3.270
	pNP-C6	175.146 ± 10.32	14.763 ± 1.09	11.864
	pNP-C8	0.171 ± 0.012	0.496 ± 0.01	0.3448
DLFae4 (A341G)	pNP-C2	5823.172 ± 221.87	44.314 ± 2.38	131.407
	pNP-C4	408.194 ± 33.86	38.897 ± 2.21	10.494
	pNP-C6	279.415 ± 18.93	10.799 ± 0.97	25.874
	pNP-C8	0.0888 ± 0.01	0.713 ± 0.01	0.1245

Note: Data are shown as mean ±standard deviations with triplicate replicate.

## Data Availability

All data generated or analyzed during this study are included in this published article and its Supplementary Information Files.

## Supplementary Materials

The following supporting information can be downloaded at: https://www.mdpi.com/article/10.3390/foods12061169/s1, Figure S1: Screening clones with enhanced activity; Figure S2: Comparison of relative enzyme activities between WT and mutants; Figure S3: Effects of pH and temperature on the activity of DLFae4 and mutants; Figure S4: Ramachandran plot of DLFae4 and mutants; Figure S5: Hydrophobic pocket graphics of DLFae4 and mutants; Figure S6: DLFae4 catalyzed the formation of insoluble phenethyl acetate; Figure S7: GC-MS analysis of the structure of the product after the reaction of vinyl acetate and 2-phenylethanol; Figure S8: HPLC analysis of the product after the reaction of vinyl acetate and cyanidin-3-O-glucoside; Table S1: Mutatable sites predicted by HotSpot wizard for hydrolytic activity of DLFae4.
