# Supplementary material for: Enhancing the Hydrolysis and Acyl Transfer Activity of Carboxylesterase DLFae4 by a Combinational Mutagenesis and In-Silico Method
Source: Foods. 2023 Mar 10;12(6):1169. doi: 10.3390/foods12061169 (PMC10048530; doi:10.3390/foods12061169)
Supplement: Supplementary file 1 [file foods-12-01169-s001.zip › foods-2169307-supplementary.pdf]

Table S1 Mutatable sites predicted by HotSpot wizard for hydrolytic activity of DLFae4

Position	Residue	Mutable	In tunnel	In catalytic pocket	Codon	Encoded AAs
145	Thr	9	√	×	VDS	Asp:1 Glu:1 Gly:2 His:1 Ile:1 Lys:1 Leu:2 Met:1 Asn:1 Gln:1 Arg:3 Ser:1 Val:2
146	Pro	8	√	×	RNS	Ala:2 Asp:1 Glu:1 Gly:2 Ile:1 Lys:1 Met:1 Asn:1 Arg:1 Ser:1 Thr:2 Val:2
132	Trp	8	√	√	VNS	Ala:2 Asp:1 Glu:1 Gly:2 His:1 Ile:1 Lys:1 Leu:2 Met:1 Asn:1 Pro:2 Gln:1 Arg:3 Ser:1 Thr:2 Val:2
157	Arg	8	√	×	NHC	Ala:1 Asp:1 Phe:1 His:1 Ile:1 Leu:1 Asn:1 Pro:1 Ser:1 Thr:1 Val:1 Tyr:1

Figure Captions

Figure S1 Screening clones with enhanced activity. (a) Screening of random mutation clones using EFA as substrate. (b) Screening of site-directed saturation mutation clones using EFA as substrate

Figure S2 Comparison of relative enzyme activities between WT and mutants

Figure S3 Effects of pH and temperature on the activity of DLFae4 and mutants. (a) The effect of temperature on activity; (b) the effect of pH on activity; (c) the effect of temperature (40°C) on thermal stability; (d) the effect of pH on stability. The maximal activity was defined as 100% and the relative activity is shown as a percentage of maximal activity. Each point presented mean ± standard deviation (n = 3)

Figure S4 Ramachandran plot of DLFae4(a) and mutants (b: DLFae4-m1; c: DLFae4-m2; d: DLFae4-m3; e: DLFae4-m4; f: DLFae4-m5). Note: The red area represented residues in the most favoured regions, the yellow area represented residues in additional regions, the light yellow area represented residues in generously allowed regions, and the white area represented residues in disallowed regions

Figure S5 Hydrophobic pocket graphics of DLFae4 (a) and mutants (b: DLFae4-m1; c: DLFae4-m2; d: DLFae4-m3; e: DLFae4-m4; f: DLFae4-m5). Note: The substrate MFA was shown in gray sticks (by element). The amino acids that differ between WT and mutants were shown by the abbreviation of amino acid and serial number. The hydrophobic amino acids that differ between WT and mutants marked with red dashed circles

Figure S6 DLFae4 catalyzed the formation of insoluble phenethyl acetate

Figure S7 GC-MS analysis of the structure of the product after the reaction of vinyl acetate and 2-phenylethanol

Figure S8 HPLC analysis of the product after the reaction of vinyl acetate and cyanidin-3-O-glucoside. Negative control: cyanidin-3-O-glucoside + enzyme, Blank control: cyanidin-3-O-glucoside + vinyl acetate + inactivating enzyme, Experimental group: cyanidin-3-O-glucoside+ vinyl acetate+ enzyme.

Figure S1

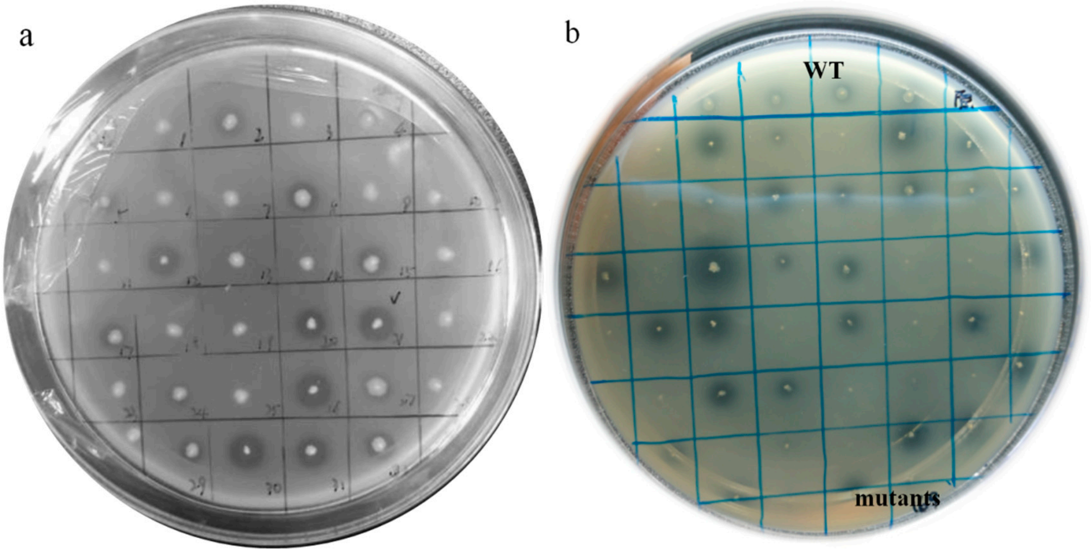

Figure S2

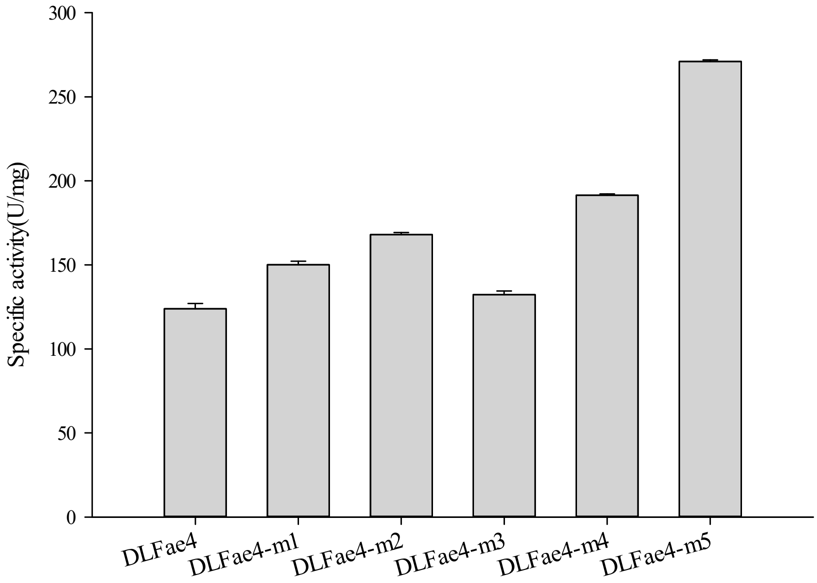
Specific activity(U/mg)
300
250
200
150
100
50
0
DLFae4
DLFae4-m1
DLFae4-m2
DLFae4-m3
DLFae4-m4
DLFae4-m5

Figure S3

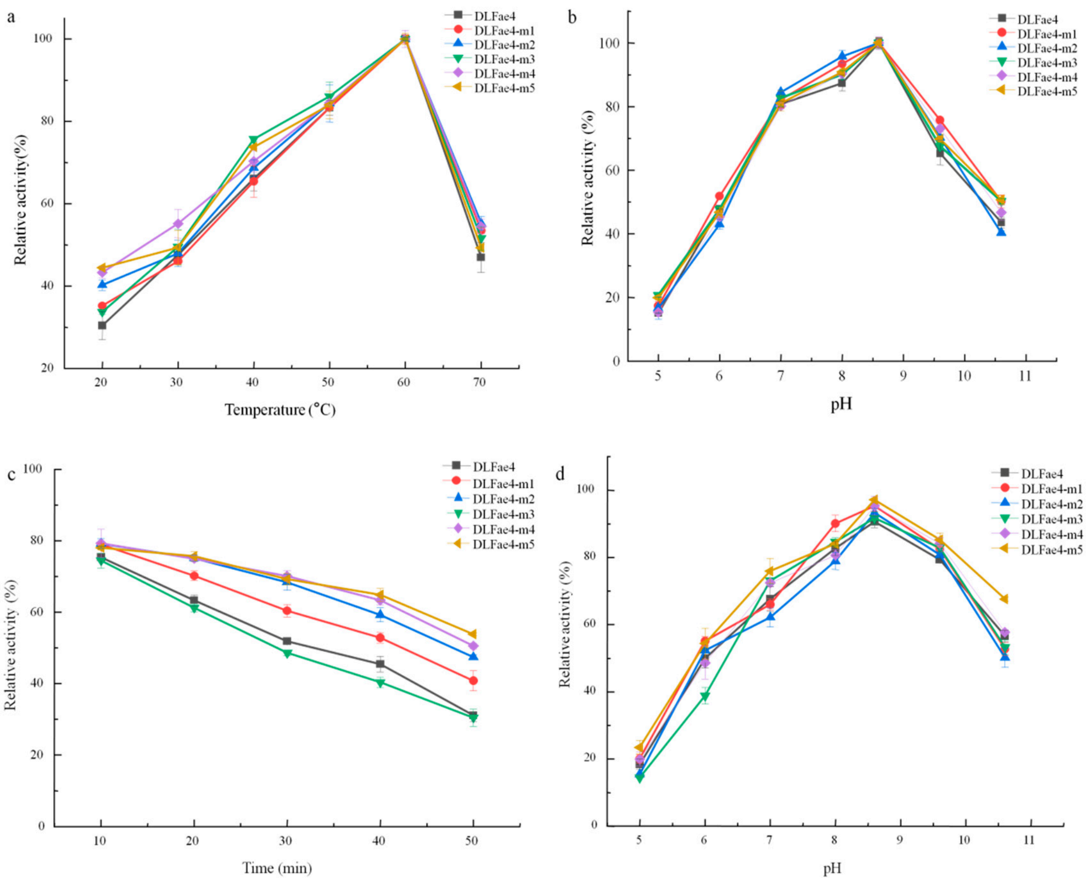

a
b
c
d
DLFae4
DLFae4-m1
DLFae4-m2
DLFae4-m3
DLFae4-m4
DLFae4-m5
Relative activity(%)
Temperature (°C)
Relative activity (%)
pH
Time (min)

Figure S4

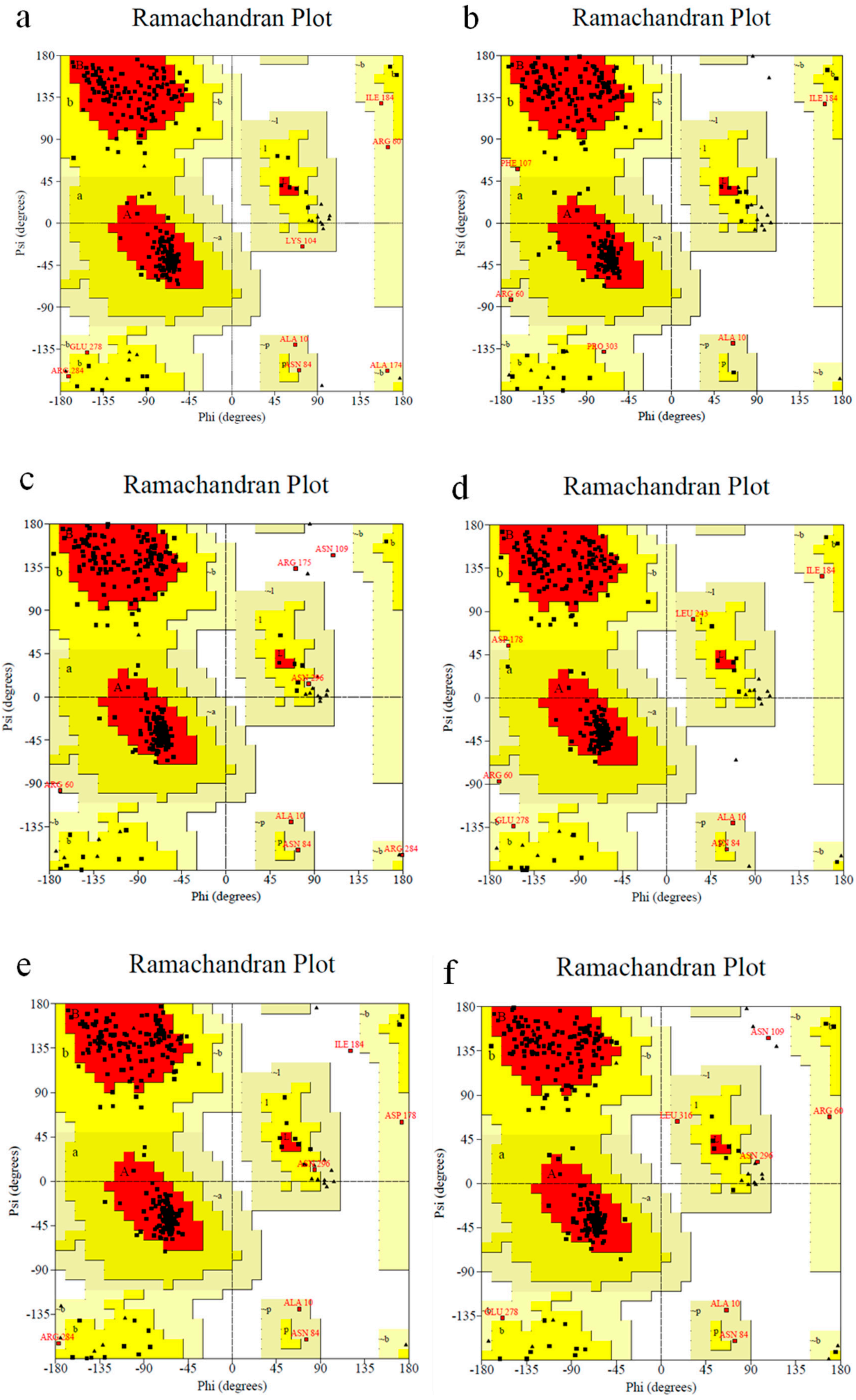
a
Ramachandran Plot
Psi (degrees)
Phi (degrees)
ILE 184
ARG 60
LYS 104
ALA 10
GLU 278
ARG 284
ASN 84
ALA 174
b
Ramachandran Plot
PHE 107
ILE 184
ARG 60
ALA 10
PRO 303
c
Ramachandran Plot
ASN 109
ARG 175
ARG 60
ALA 10
ASN 84
ARG 284
d
Ramachandran Plot
ILE 184
LEU 243
ASP 178
ARG 60
GLU 278
ALA 10
e
Ramachandran Plot
ILE 184
ASP 178
ALA 10
ASN 84
ARG 284
f
Ramachandran Plot
ASN 109
ARG 60
LEU 316
ASN 296
GLU 278
ALA 10
ASN 84

Figure S5

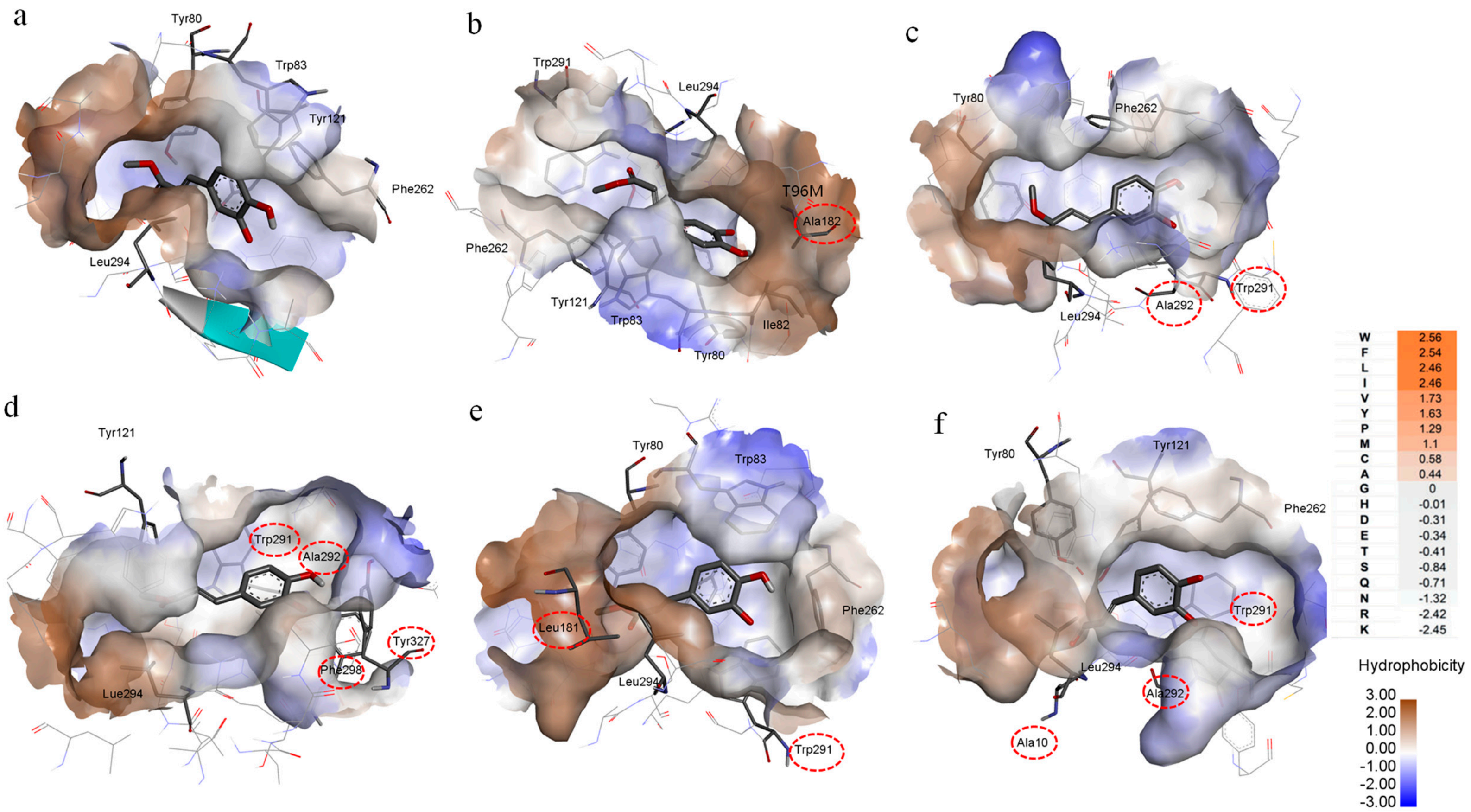

a
Tyr80
Trp83
Tyr121
Phe262
Leu294
b
Trp291
Leu294
T96M
Ala182
Phe262
Tyr121
Trp83
Ile82
Tyr80
c
Tyr80
Phe262
Trp291
Ala292
Leu294
d
Tyr121
Trp291
Ala292
Tyr327
Phe298
Lue294
e
Tyr80
Trp83
Phe262
Leu181
Leu294
Trp291
f
Tyr80
Tyr121
Phe262
Trp291
Leu294
Ala292
Ala10
W 2.56
F 2.54
L 2.46
I 2.46
V 1.73
Y 1.63
P 1.29
M 1.1
C 0.58
A 0.44
G 0
H -0.01
D -0.31
E -0.34
T -0.41
S -0.84
Q -0.71
N -1.32
R -2.42
K -2.45
Hydrophobicity
3.00
2.00
1.00
0.00
-1.00
-2.00
-3.00

Figure S6

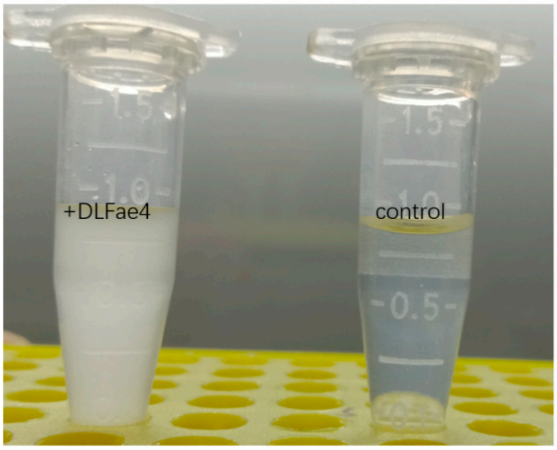

Figure S7

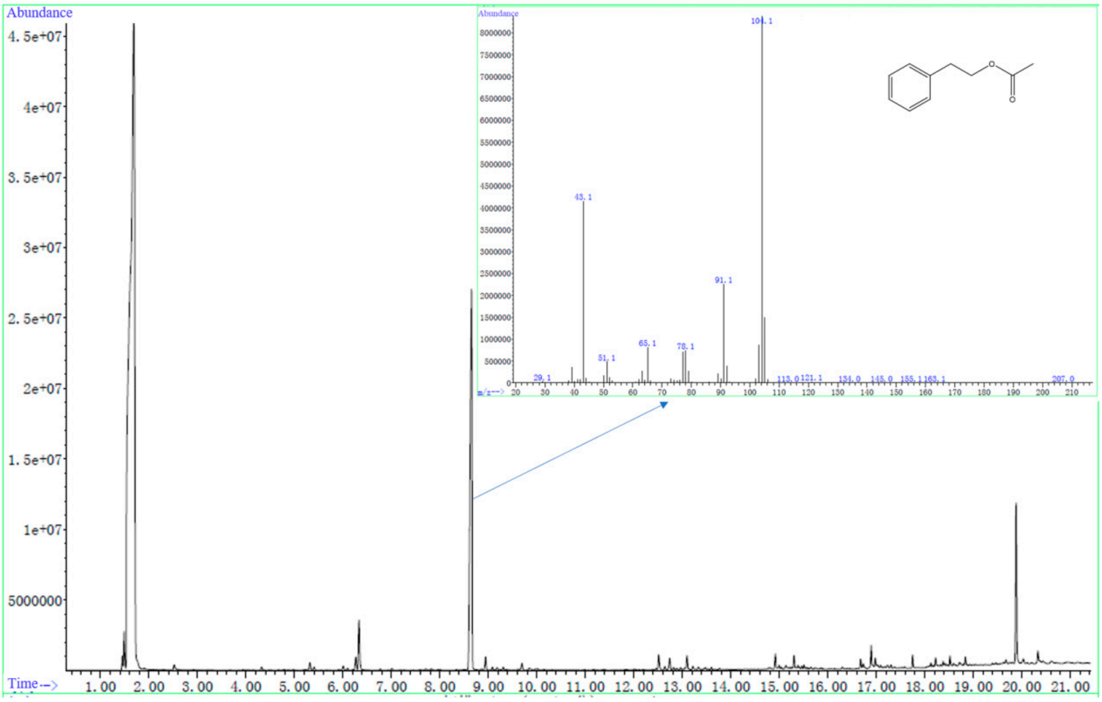
Abundance
4.5e+07
4e+07
3.5e+07
3e+07
2.5e+07
2e+07
1.5e+07
1e+07
5000000
Time-->
1.00 2.00 3.00 4.00 5.00 6.00 7.00 8.00 9.00 10.00 11.00 12.00 13.00 14.00 15.00 16.00 17.00 18.00 19.00 20.00 21.00
Abundance
104.1
43.1
91.1
65.1
78.1
51.1
29.1
113.0
121.1
134.0
145.0
155.1
163.1
207.0
m/z-->
O

Figure S8

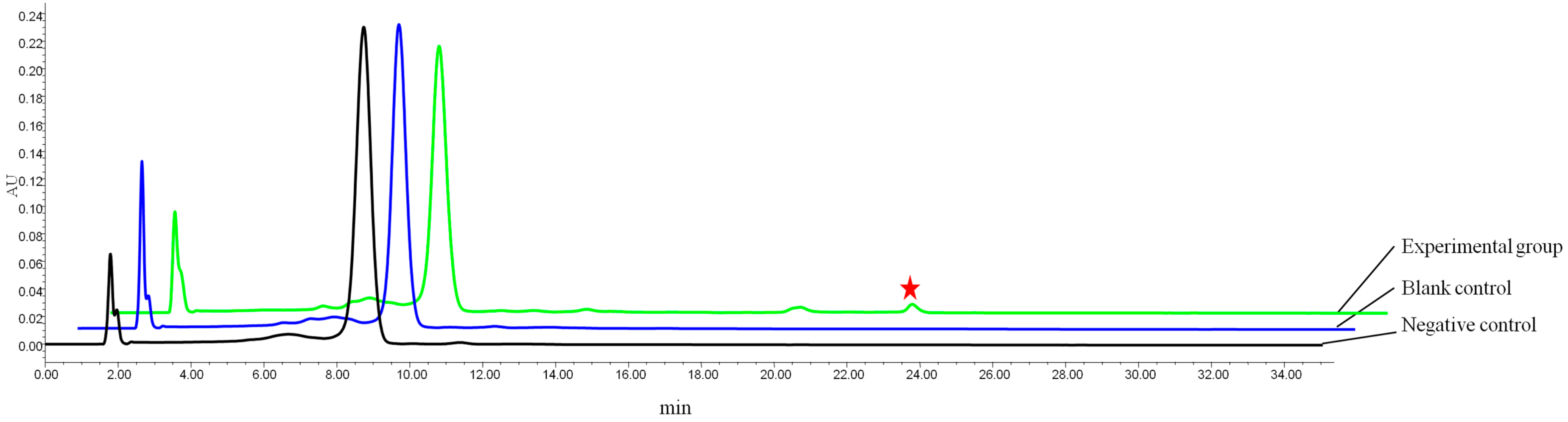

0.24
0.22
0.20
0.18
0.16
0.14
0.12
0.10
0.08
0.06
0.04
0.02
0.00
AU
0.00
2.00
4.00
6.00
8.00
10.00
12.00
14.00
16.00
18.00
20.00
22.00
24.00
26.00
28.00
30.00
32.00
34.00
min
Experimental group
Blank control
Negative control
